# A systematic review of the efficacy of repetitive transcranial magnetic stimulation in treating dysarthria in patients with Parkinson's disease

**DOI:** 10.3389/fnagi.2025.1501640

**Published:** 2025-02-05

**Authors:** Kerong Chen, Sitong Zhou, Shiyu Lu, Yuliang Qin, Xinyao Li, Yi Li, Tianyun Liu, Mei Zhang, Kun Xu, Hongping Shi, Xiaoman Lv, Kai Yuan, Hongling Shi, Dongdong Qin

**Affiliations:** ^1^Department of Rehabilitation Medicine, The Third People's Hospital of Yunnan Province, Kunming, Yunnan, China; ^2^Key Laboratory of Traditional Chinese Medicine for Prevention and Treatment of Neuropsychiatric Diseases, Yunnan University of Chinese Medicine, Kunming, Yunnan, China; ^3^The People's Hospital of Mengzi, The Affiliated Hospital of Yunnan University of Chinese Medicine, Mengzi, Honghe, China; ^4^Second Clinical Medical College, Yunnan University of Chinese Medicine, Kunming, Yunnan, China

**Keywords:** Parkinson's disease, dysarthria, repetitive transcranial magnetic stimulation, efficacy, systematic review

## Abstract

**Objective:**

To analyze the literature on the efficacy of repetitive transcranial magnetic stimulation (rTMS) in treating dysarthria in patients with Parkinson's disease (PD) and provide a reference for targeted clinical treatment of dysarthria in PD patients.

**Methods:**

A systematic search was conducted in English and Chinese databases, including Embase, Cochrane, Medline, PubMed, CNKI, Wanfang, Chinese Biomedical Literature Database, and VIP Database, for relevant literature on rTMS treatment for dysarthria in PD patients. The search timeframe was from the inception of each database to October 2023. Literature was screened according to inclusion and exclusion criteria. Two researchers extracted information on study subjects, age, intervention methods, intervention duration, intervention frequency, evaluation indicators, and intervention results from the included literature. The modified Jadad scale was used to evaluate the quality of the literature.

**Results:**

A total of seven studies were included, mainly focusing on the frequency, duration, and stimulation site of rTMS for dysarthria in PD patients. Six studies indicated that rTMS treatment improved dysarthria in PD patients.

**Conclusion:**

Repetitive transcranial magnetic stimulation has a positive effect on improving dysarthria in PD patients, but further research is needed to determine its efficacy.

## 1 Introduction

Parkinson's disease (PD) is a common progressive neurodegenerative disorder frequently encountered in clinical practice. Its primary pathological features include the progressive loss of dopaminergic neurons in the substantia nigra pars compacta and the formation of Lewy bodies. Genetic and epidemiological studies indicate that PD results from the interplay of multiple factors, including genetics, environmental factors, and neurological aging (Bloem et al., 2021). Epidemiological surveys reveal that the prevalence of PD in Western countries reaches 2%−3% among individuals aged 65 years and older and exceeds 4% in those aged 80 years and above (Simon et al., 2020). A community-based study in China showed that the prevalence of PD among the elderly population aged 60 years and older was 1.37% [95% CI (1.02%, 1.73%)], with an estimated total of 3.62 million PD patients in China (Qi et al., 2021). As the world's most populous country, the number of PD patients in China is projected to reach 5 million by 2030, accounting for nearly half of the global PD patient population (Parkinson's Disease Movement Disorders Group Neurologist Branch of Chinese Medical Doctor Association, 2020).

Research has demonstrated that dysarthria is present in 70%−90% of PD patients in the early stages of the disease and even in the prodromal phase (prodromal PD, pPD), making it one of the most common symptoms of PD (Ma et al., 2014; Schalling et al., 2017; Collaborators, 2019; Fereshtehnejad et al., 2019). Dysarthria in PD is primarily characterized by abnormalities in the force, speed, range, amplitude, stability, or accuracy of the movements required for speech production, including respiration, phonation, resonance, and rhythm. Clinical manifestations mainly include lingual muscle rigidity and tremors, leading to reduced mouth opening, imprecise lip closure, monotonous intonation, short sentences, reduced stress, slurred speech, and either excessively fast or slow speech rate. These symptoms significantly impact patients' communication abilities, resulting in reduced social interaction and diminished quality of life (Li and Li, 2021; Lu et al., 2022). Currently, commonly used dysarthria training methods include oral motor exercises, articulatory movement and speech ability training, as well as voice and breathing exercises. However, high-quality evidence supporting the efficacy of these methods remains limited (Han, 2019), and clinical treatment often requires prolonged periods with suboptimal outcomes.

Repetitive transcranial magnetic stimulation (rTMS) is a non-invasive neuromodulation technique that delivers repetitive stimulation to specific areas of the cerebral cortex using pulsed magnetic fields (Lin et al., 2020). High-frequency rTMS (≥5 Hz) excites neurons, increasing local cortical excitability, regional cerebral blood flow, and metabolism (Elahi et al., 2009). While, low-frequency rTMS ( ≤ 1 Hz) inhibits neurons, thereby modulating cortical excitability and central nervous system plasticity (Du et al., 2019). rTMS has a regulatory effect on both local and remote neural functions (Brandt et al., 2021). Due to its non-invasive, effective, and cost-efficient nature, rTMS has emerged as a promising rehabilitation technology with widespread applications in the treatment and neurorehabilitation of various neurological and psychiatric disorders, such as neuropathic pain, severe depression, post-acute stroke motor dysfunction, epilepsy, cerebrovascular diseases, and Parkinson's disease (PD) (Ma et al., 2020). In the context of PD, rTMS is primarily employed to alleviate motor symptoms, depression, cognitive dysfunction, and other associated impairments (Chi, 2020).

The present study aims to systematically evaluate the clinical efficacy of rTMS in treating dysarthria in PD patients, with the ultimate goal of providing evidence-based guidance for the clinical application of rTMS in the management of PD-related dysarthria.

## 2 Materials and methods

### 2.1 Search strategy

A comprehensive literature search was conducted in EMBASE, Cochrane, Medline, PubMed, China National Knowledge Infrastructure (CNKI), Wanfang Database, Chinese Biomedical Literature Database, and VIP Database. The search terms were divided into three groups: (1) population: Chinese search terms included “Parkinson's disease,” “idiopathic Parkinson's disease,” “Lewy body Parkinson's disease” and “primary parkinsonism.” English search terms included “Parkinson's disease,” “idiopathic Parkinson's disease,” “Lewy Body Parkinson's disease,” “Parkinson's disease,” “idiopathic Parkinson's disease,” “primary Parkinsonism,” and “paralysis agitans;” (2) symptoms: Chinese search terms included “dysarthria,” “voice disorders,” “hypokinetic dysarthria,” and “language disorders.” English search terms included “dysarthria,” “hypokinetic dysarthria,” and “language disorders;” (3) intervention: the Chinese search term was “repetitive transcranial magnetic stimulation” and English search terms included “repetitive transcranial magnetic stimulation,” “rTMS,” “transcranial magnetic stimulation,” and “noninvasive brain stimulation.” The entire search process was completed in December 2023.

### 2.2 Inclusion and exclusion criteria

Inclusion criteria: (1) studies focusing on dysarthria in Parkinson's disease; (2) studies involving rTMS as an intervention; (3) studies with assessments including scales and test indicators; (4) studies published in Chinese or English. Exclusion criteria: (1) full text not available; (2) duplicate publications; (3) lack of relevant measurement indicators; (4) review articles.

### 2.3 Literature screening and inclusion

Literature screening and data extraction were conducted independently by two researchers following predetermined criteria. Any disagreements were resolved through discussion or, if necessary, adjudication by a third party. EndNote software was used to manage the references. Initial screening involved reviewing titles and abstracts, and full-text reviews were performed for studies that potentially met the inclusion criteria to confirm eligibility. Data were extracted and organized using Excel and included information on study participants, age, intervention methods, duration and frequency of interventions, evaluation indicators, and intervention outcomes.

### 2.4 Quality assessment of included studies

The quality assessment of all included studies was performed by two researchers. The methodological quality of the included studies was evaluated using the simple assessment method recommended by the Cochrane Collaboration. The evaluation criteria included key indicators of internal validity, such as the correctness of randomization methods, the correctness of allocation concealment, the use of intention-to-treat analysis, the implementation of blinding, the reporting of dropouts and withdrawals, and the comparability of baseline data.

Studies were rated as follows:

- Grade A: all quality assessment criteria were met, indicating the lowest possibility of bias.
- Grade B: one or more of the quality assessment criteria were only partially met, indicating a moderate possibility of corresponding bias.
- Grade C: one or more of the quality assessment criteria were not met at all, indicating a high possibility of bias.

After independently assessing the quality of the literature, the two researchers discussed the quality of each study according to the above evaluation criteria and reached a consensus on the final decision to include or exclude the study.

### 2.5 Data extraction

After reading the full text, the following data were extracted: (1) inclusion criteria and sample size; (2) sampling and grouping methods and processes; (3) basic information of the study subjects; (4) study conditions; (5) rTMS treatment device type; (6) parameter settings, including frequency, intensity, location, number of sessions (sequences), duration, and treatment compliance; and (7) means and standard deviations of continuous outcome measures.

## 3 Results

### 3.1 General characteristics of included studies

**Figure 1:**
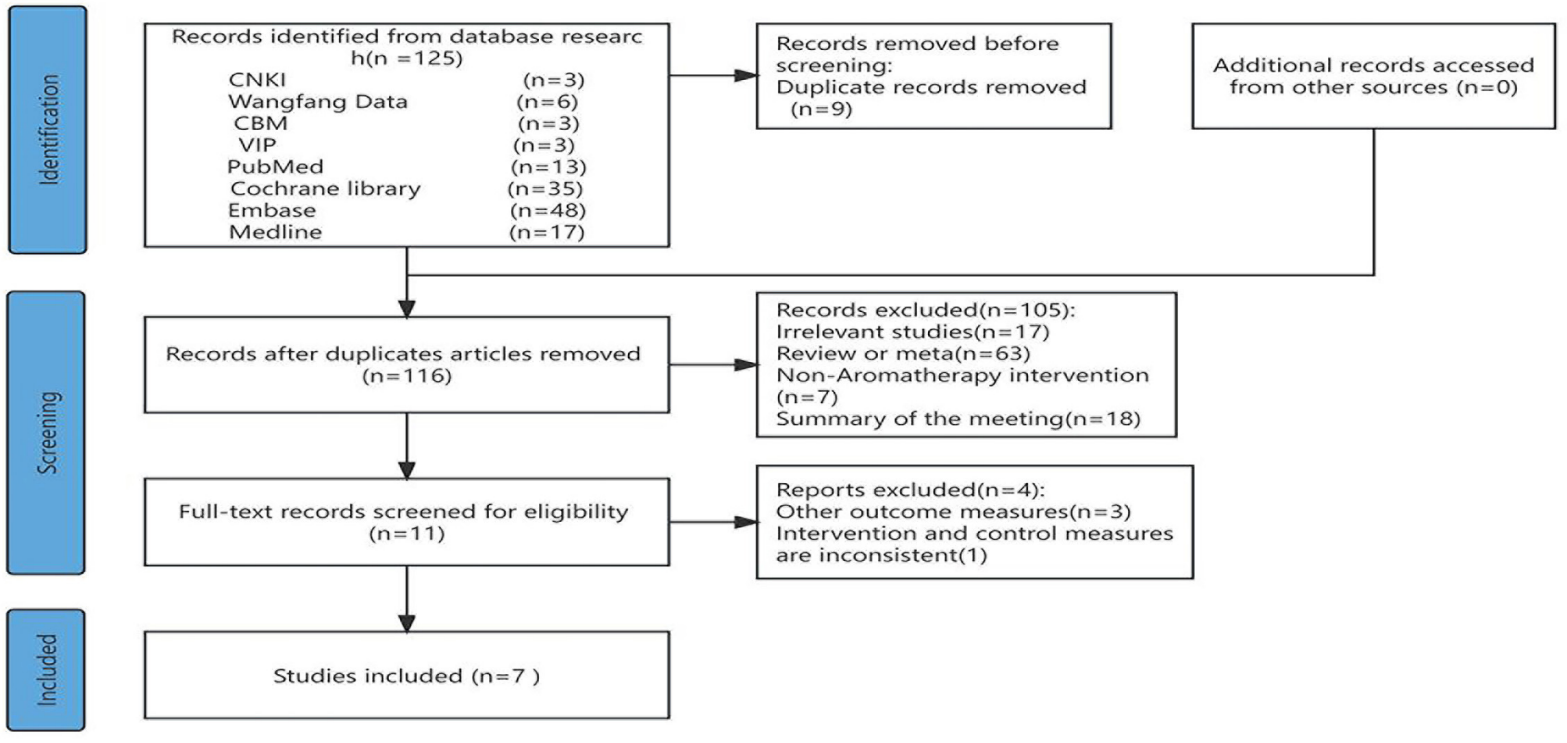
PRISMA flow diagram of the search process for studies.

The initial search yielded 125 relevant studies, including 15 in Chinese and 110 in English. After excluding 88 studies due to duplicate publication, overlap, or obvious non-compliance with the inclusion criteria, and further excluding case-control and descriptive studies based on title and abstract screening, 37 clinical controlled studies were included. By retrieving full texts, reading, and conducting quality assessments, 30 non-randomized controlled trials and clinical trials without a control group were excluded. Finally, seven studies were included, comprising two in Chinese and five in English. The literature screening process and results are presented in Figure 1.

### 3.2 Characteristics of included studies

Among the seven included studies, two were published in Chinese (Wang et al., 2022a,b), and five were published in English (Hartelius et al., 2010; Eliasova et al., 2013; Brabenec et al., 2019, 2021; Gómez-Rodellar et al., 2023). The basic characteristics of the included studies are summarized in Table 1.

**Table 1:** Basic characteristics of included literature.

**Study**	**Mean age**		**Disease duration**		**Sample size**		**Intervention**		**Intervention duration**	**Outcome measures**	**Results**	**Conclusion**
	**Experimental group**	**Control group**	**Experimental group**	**Control group**	**Experimental group**	**Control group**	**Experimental group**	**Control group**				
Brabenec et al. (2019)	67.21 ± 6.18	67.21 ± 6.18	6.81 ± 5.00	6.81 ± 5.00	16^#^	16^#^	10 Hz and/or 1 Hz rTMS stimulation over the left orofacial primary motor area, the right superior temporal gyrus (STG)	rTMS stimulation over the vertex	Five times in 2 weeks (2,250 pulses/session for 10 Hz stimulations and 1,800 pulses/session for 1 Hz stimulations)	Acoustic parameters of speech prosody and articulations; reading task fMRI and rfMRI	The 1 Hz STG stimulation led to considerable enhancements in the relative standard deviation of the second formant. Additionally, low-frequency rTMS exhibited significant behavioral effects that exceeded those elicited by rTMS at the control stimulation site	Improved articulation, activation of the right STG, as well as enhanced rs-FC between the right STG and the cortical region involved in an overt speech control
Brabenec et al. (2021)	71.70 (IQR 62.20–75.50)	71.50 (IQR 60.90–78.00)	4.00 (IQR 2.00–10.50)	3.00 (IQR 1.00–8.20)	20	13	1 Hz rTMS stimulation over the right STG and OFSM1	Sham stimulation	40 min per session, five times per week, 10 sessions completed within 2 weeks (1,800 pulses/session)	The 3F Test–Dysarthric Profile (3FT); reading task fMRI and rfMRI	Real stimulation, as opposed to sham stimulation, resulted in heightened activation within the orofacial sensorimotor cortex and the caudate nucleus, along with improved intrinsic connectivity between these regions and the targeted area. In the real rTMS cohort, the relative change in phonetic scores surpassed that recorded in the sham group	Significant enhancement of 3FT phonetic scores, activation of the OFSM1 and caudate nucleus, as well as increased intrinsic rs-FC of these regions
Eliasova et al. (2013)	64.58 ± 8.04	64.00 ± 8.55	10.75 ± 7.48	10.75 ± 7.48	12	21	10 Hz rTMS stimulation over the dominant (left) orofacial primary motor area (SM1) and over the left dorsolateral prefrontal cortex (DLPFC)	Regular dopaminergic treatment	30 min per session, and the stimulation was performed on day 1 and day 3 (2,250 pulses/session)	The 3F Test–Dysarthric Profile (3FT) and acoustic analysis	Both the rate of speech and its quality exhibited an enhancement of ~21%. In the reading sentences assessment, the average Harmonics-to-Noise Ratio (HNR) values demonstrated an increase of roughly 15%. Regarding the vowel task, the Vowel Space Area (VSA) values escalated by ~79%	Improvement in voice quality and intensity and increases in speech rate and tongue movements
Gómez-Rodellar et al. (2023)	69.6 ± 8.07	70.29 ± 6.47	/	/	9	9	1 Hz rTMS stimulation over the right posterior and superior temporal gyrus	Sham stimulation	40 min per session, five times per week, for a total of 2 weeks (1,800 pulses/session)	Acoustic analysis	The rTMS demonstrated a notable inter-speaker influence on articulation, prosody, and intelligibility, as assessed by the III. Phonetics subtest	Significant improvements in phonation instability
Hartelius et al. (2010)	57.00 ± 8.90	57.00 ± 8.90	3.60 ± 2.40	3.60 ± 2.40	10^#^	10^#^	10 Hz rTMS stimulation over motor hand area contralateral to the more severely affected upper limb	Placebo stimulation	Four blocks of 20-train 10 Hz rTMS (train duration: 2.5 s; inter-train intervals: 5 s, 2,000 pulses/session)	Acoustic analysis	The findings revealed no statistically significant variations among the conditions regarding the duration of sustained fricative or vowel phonation, diadochokinetic rates, or levels of intelligibility	No significant differences between any of the conditions regarding duration of sustained fricative or sustained vowel phonation, diadochokinetic rates or intelligibility
Wang et al. (2022a)	68.90 ± 2.90	73.80 ± 4.50	4.50 ± 3.40	3.20 ± 2.70	10	10	5 Hz rTMS stimulation over bilateral M1	Conventional articulation training	Each side for 10 min, 20 min/session, once a day, lasting a total of 30 days	Frenchay dysarthria assessment scale; articulation and phonetics measurement; voice and speech disorder detection and correction	After 30 days of intervention, the experimental cohort exhibited marked improvements in Frenchay scores, articulation clarity, sustained speech capacity (speech rate), oral alternating velocity, and amplitude standard deviation relative to the control cohort	Significant improvement in the articulatory motor function, speech intelligibility, loudness control, and speech rate control
Wang et al. (2022b)	69.53 ± 8.41	72.53 ± 6.04	5.13 ± 1.22	4.13 ± 1.05	15	15	5 Hz rTMS stimulation over bilateral M1	Conventional medication and voice training	Each side for 10 min, 20 min/session, once a day, lasting a total of 30 days	Voice function assessment and voice handicap index (VIH) questionnaire	In comparison to the control group, the experimental group demonstrated significantly increased metrics in Maximum Phonation Time (MPT), continuous Maximum Phonation Capacity (cMCA), fundamental frequency (F0t), and energy coefficient (Ec) following treatment. The impact of rTMS on respiratory support, the synchronization of respiration and phonation, vocal fold tremor, and harmonic energy decay during vocal fold oscillation was particularly more evident in patients diagnosed with PD	Improvement in the respiratory support ability, the coordination ability of breathing and vocalization, the regularity of vocal cord closure, and the forward movement ability of the tongue

The symbol of ^#^ means that the experimental and control groups consisted of the same participants. rTMS, repetitive transcranial magnetic stimulation; RMT, resting motor threshold; rs-FC, resting state functional connectivity; OFSM1, orofacial sensorimotor cortex; SM1, the primary orofacial sensorimotor area; M1, the primary motor area; PD, Parkinson's disease.

### 3.3 Quality assessment of included studies

The quality of the included randomized controlled trials was assessed using the evaluation criteria from the Cochrane Collaboration Handbook, version 5.1.0. The results showed that all seven studies were rated as grade B in terms of quality, as detailed in Table 2.

**Table 2:** Quality assessment of included studies.

**Included study**	**Random sequence generation**	**Allocation concealment**	**Blinding**		**Incomplete outcome data**	**Selective reporting**	**Other bias**	**Study quality**
			**Intervention**	**Outcome assessor**				
Brabenec et al. (2019)	Low	Low	High	High	Low	Low	Low	B
Brabenec et al. (2021)	Low	Unclear	High	High	Low	Low	Low	B
Eliasova et al. (2013)	Unclear	Unclear	High	High	Low	Low	Low	B
Gómez-Rodellar et al. (2023)	Low	Unclear	High	High	Low	Low	Low	B
Hartelius et al. (2010)	Low	Unclear	High	High	Low	Low	Low	B
Wang et al. (2022a)	Low	Unclear	High	High	Low	Low	Low	B
Wang et al. (2022b)	Low	Unclear	High	High	Low	Low	Low	B

This study conducted a comprehensive evaluation of rTMS in the treatment of dysarthria in Parkinson's disease (PD) to assess its therapeutic efficacy. rTMS produces brief pulse stimulation in a non-invasive manner, generating induced currents in the cortex that lead to axonal depolarization and action potential generation in neurons (Iglesias, 2020). Common stimulation targets for rTMS in the treatment of speech disorders in PD include the primary motor cortex (M1), superior temporal gyrus (STG), and dorsolateral prefrontal cortex (DLPFC).

In this systematic review, seven included studies utilized the aforementioned stimulation sites, strictly adhering to the inclusion and exclusion criteria. However, the analysis results may still contain some errors. The number of included studies was relatively small, and the quality of the literature varied. The evaluation methods and indicators were not uniform across the included studies, making it difficult to weigh the effectiveness of the literature during the analysis process, and high-quality studies were scarce. Furthermore, there were differences in the methods of the included studies, such as insufficient use of randomization methods and inconsistencies in outcome measurement methods. These differences and limitations may lead to serious bias in the study results. Therefore, after data extraction, a quantitative analysis could not be performed, and a descriptive systematic review method was used to analyze the effects of rTMS on speech function in PD patients with dysarthria. Future research should increase the sample size, establish unified analysis methods, include more high-quality studies, exclude low-quality studies, and conduct long-term follow-ups on clinical samples. This will provide high-quality, reliable, and valid data for an effective systematic review.

## 4 Discussion

### 4.1 Quality assessment of included studies

The methodological quality of the studies included in this research was graded as level B. Most studies did not specify whether allocation concealment and assessor blinding were performed. Due to the nature of the intervention, it is challenging to achieve blinding of study participants, which may lead to certain biases in the outcome measures. Moreover, this study only searched Chinese and English databases. As there have been relatively few relevant publications in recent years, both domestically and internationally, only seven articles were ultimately included in this study, with five in English and two in Chinese. Gray literature and studies published in other languages were not considered, which may result in a degree of publication bias.

### 4.2 Mechanism of rTMS in the treatment of Parkinsonian dysarthria

**Figure 2:**
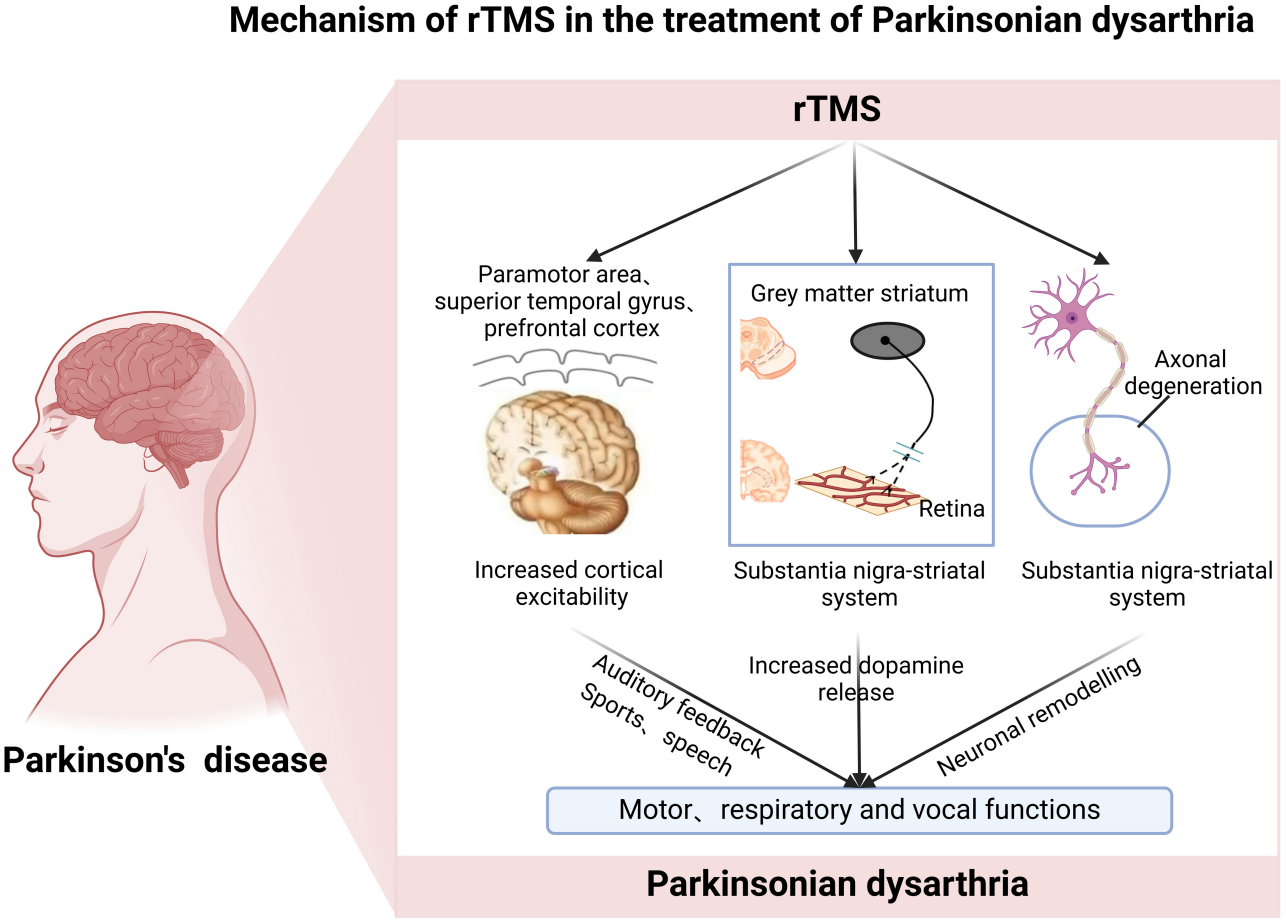
Roles and mechanisms of rTMS in the treatment of Parkinsonian dysarthria.

Dysarthria is a speech disorder caused by paralysis, weakness, or incoordination of speech-related muscles due to central or peripheral nerve damage. Speech production requires mapping phonological representations onto the articulation network, and accurate speech output depends on the auditory-motor integration process mediated by the dorsal language pathway (Wang et al., 2023). Specifically, the cortical regions involved in auditory-motor integration include the superior temporal gyrus (STG), primary motor cortex (M1), dorsolateral prefrontal cortex (DLPFC), and premotor cortex, which are closely related to the occurrence of PD speech disorders (Li et al., 2022; Steurer et al., 2022; Wang et al., 2023). Studies have demonstrated that rTMS can induce an increase in dopamine levels in the nigrostriatal system (Khedr et al., 2007). Research on rodents has indicated that rTMS can effectively reduce the loss of dopaminergic neurons in the nigrostriatal pathway and improve cognitive and motor functions (Zong et al., 2022). Current evidence suggests that low-frequency rTMS ( ≤ 1 Hz) has an inhibitory effect on target neurons, which helps to reduce excessive neural activity that may lead to Parkinsonian dysarthria and other motor symptoms (Zhang et al., 2022). Dysarthria in PD patients is primarily attributed to impaired activation and coordination of speech-related muscles, resulting from the progressive degeneration of the nigrostriatal pathway (Dashtipour et al., 2018). Additionally, reduced excitability of the basal ganglia to the cortex leads to physiological deficits in the larynx, joints, and respiratory system, as well as decreased cortical activity in areas of the speech production network (Narayana et al., 2022). Speech disorders in PD patients may also result from damage to the central or peripheral nerves, causing dysfunctions in the muscle movements involved in speech production (Iglesias, 2020). However, the brain's neural pathways possess plasticity, enabling the establishment of new pathways through stimulation of the neural network. This plasticity can promote the recovery of motor function in the organs of articulation, thus enhancing speech fluency and normalization in patients with dysarthria (Zhu et al., 2019). High-frequency rTMS enhances cortical excitability and modulates neural activity in specific brain regions involved in motor control and speech production, such as the supplementary motor area (SMA) and prefrontal cortex, and can be used to treat dysarthria (Brabenec et al., 2021). rTMS enhances brain neuroplasticity by modulating synaptic activity through both long-duration excitation and inhibition. It regulates neurotransmitter and neuromodulator levels, affects ion channels, and alters the expression of genes involved in plasticity. These effects promote the reactivation of the classical brain network and recruit underactive brain regions surrounding the lesion, ultimately improving speech and language function and accelerating speech recovery. Through functional reorganization, rTMS facilitates cortical remodeling (Hoogendam et al., 2010; Murdoch and Barwood, 2013; Wang et al., 2015; Valero-Cabré et al., 2017; Zhou and Dan, 2021). Additionally, rTMS has been shown to improve cerebral blood flow and metabolism, regulate ionic balance, inhibit apoptosis, and induce persistent changes in cortical excitability. It also affects neurotransmitter release and activates gene and protein expression, further enhancing cortical functional remodeling (Wang F. et al., 2022). By modulating the excitability of the cerebral cortex, rTMS can control motor function in the mouth, face, and tongue, thereby improving the motor function necessary for speech and language production. Therefore, different frequencies of rTMS can be utilized to regulate brain excitability, activate the nigrostriatal pathway, and increase levels of neurotransmitters such as dopamine, thereby improving the motor and coordination functions of muscle groups related to movement, respiration, and phonation, ultimately ameliorating dysarthria (Figure 2).

### 4.3 Analysis of results

In this study, a total of seven articles (Hartelius et al., 2010; Eliasova et al., 2013; Brabenec et al., 2021; Wang et al., 2022a,b; Gómez-Rodellar et al., 2023) were included to explore the clinical efficacy of rTMS for Parkinsonian dysarthria. Among them, two randomized controlled trials (RCTs) (Eliasova et al., 2013; Brabenec et al., 2021) that included the 3F Test–Dysarthric Profile (3FT—subtests of facial dynamics, phonation, and phonetics) as outcome measures showed that rTMS can improve symptoms of Parkinsonian dysarthria, such as insufficient airflow, frequency/amplitude perturbation, and impaired speech rate and rhythm, to a certain extent.

One RCT that measured outcomes using the Frenchay Dysarthria Assessment indicated that rTMS can improve articulation movements, articulation clarity, ability to control loudness (Wang et al., 2022b), and ability to control speech rate in Parkinsonian dysarthria.

Two other studies used speech and voice as outcome measures. The results showed that rTMS can enhance respiratory support, coordination of respiration and phonation, and the regularity of vocal fold closure in patients with Parkinsonian dysarthria.

Previous evidence has demonstrated that rTMS is beneficial for improving prosody, loudness, and rhythm, enhancing speech function (Brabenec et al., 2021; Li et al., 2023), and significantly increasing voice quality, loudness, and vocal intensity, as well as tongue flexibility and range of motion in patients with Parkinsonian dysarthria. These findings are consistent with the results of the current study (Wang et al., 2022b). However, Li T showed that low-frequency rTMS cannot significantly improve comprehension and repetition abilities in patients with aphasia (Li et al., 2020), and DIAS (Dias et al., 2006) and ELIASOVA et al. (Lerner et al., 2019). found that the application of rTMS in patients with Parkinsonian language disorders did not result in significant changes in acoustic assessment indicators (fundamental frequency and vocal intensity). These findings are inconsistent with the aforementioned results, and the discrepancies may be attributed to factors such as differences in stimulation sites. To determine whether rTMS can improve language function in patients with Parkinsonian dysarthria, more high-quality studies need to be included for analysis in the future.

### 4.4 The curative effect of rTMS on dysarthria post PD

rTMS, as a non-invasive brain stimulation technique, has shown potential for improving dysarthria in patients with PD. Studies indicate that many patients experience improvements in speech intelligibility and fluency following rTMS treatment. However, findings regarding its long-term efficacy and durability are inconsistent. Some patients maintain improved speech function for several months, while others experience symptom relapse within a shorter time.

Among the seven studies included, the duration of efficacy observation ranged from 1 to 10 weeks. Longer treatment durations appeared to yield better outcomes in language function for PD patients. For instance, L. Hartelius et al. conducted a 1-week rTMS intervention with 10 PD patients and found no significant differences in fricative duration, sustained vowel articulation, or mediated motor rate compared to pre-treatment levels (Hartelius et al., 2010). In contrast, a 10-week study by Lubos Brabenec et al. demonstrated that functional improvements in language, including articulation accuracy, spontaneous speech, reading, bimanual motor tasks, intonation quality, and speech intelligibility, which persisted for at least 8 weeks post-treatment (Brabenec et al., 2019, 2021). Additionally, Xiaowen Wang's 30-day rTMS treatment study showed further improvements in voice function compared to the follow-up period (Eliasova et al., 2013).

### 4.5 Safety and feasibility of rTMS treatment

In clinical applications, adverse reactions to rTMS include seizures, headaches, nausea, and vomiting. Among these adverse reactions, seizures are the most severe, and research has shown that this reaction is mostly induced by high-frequency stimulation (Li et al., 2015; Shah-Basak et al., 2016; Bucur and Papagno, 2019; Duncan et al., 2020; Li et al., 2020). Therefore, special attention should be paid to the safety of rTMS during its clinical application. In this study, a total of seven articles were included, and none of them reported adverse events related to the use of rTMS treatment. rTMS treatment was generally well-tolerated in the seven studies included in this paper. Of these, three studies explicitly reported the absence of adverse effects (Hartelius et al., 2010; Eliasova et al., 2013; Brabenec et al., 2021), while the remaining four studies did not provide details regarding the presence or absence of adverse effects associated with rTMS (Wang et al., 2015; Brabenec et al., 2019; Wang F. et al., 2022; Gómez-Rodellar et al., 2023). In all the included studies, most patients were willing to receive rTMS stimulation treatment. The rTMS treatment provided in each study was easy to use, had good operability, and patients had a high acceptance of the treatment. However, due to the limited number of studies included in this review, the safety of rTMS in the treatment of Parkinsonian dysarthria requires further investigation.

### 4.6 Differences in research content on rTMS treatment for patients with Parkinsonian dysarthria

This study preliminarily confirms that rTMS has a certain effect on improving patients' articulation function, which brings positive influences on the prevention and symptom intervention for patients with Parkinsonian dysarthria. However, considering the included studies, there are differences in the intervention protocols, suggesting that the intervention plans for rTMS treatment in patients with Parkinsonian dysarthria need further improvement. Due to the differences in assessment tools and expression methods, it is difficult to conduct a combined analysis of the intervention effects of rTMS on related symptoms in patients with Parkinsonian dysarthria. This may be because there is currently no specific assessment tool for Parkinsonian dysarthria. Moreover, there are limited studies on the application of rTMS treatment in patients with Parkinsonian dysarthria, resulting in a small amount of data obtained, which further limits data pooling and quantitative analysis.

## 5 Conclusion

This systematic review demonstrates that rTMS treatment for Parkinsonian dysarthria is safe and feasible, and has a certain effect on intervening related symptoms of Parkinsonian dysarthria, providing a potentially effective intervention method for clinical treatment of Parkinsonian dysarthria. However, this study has certain limitations. Firstly, the sample size of the included literature is small and limited to Chinese and English literature, which may lead to publication bias. Secondly, there are differences in the assessment scales among the literature, so a large number of effect size combined analyses could not be performed, which may affect the research results. Thirdly, in the included studies, the assessors did not implement blinding when evaluating patients, which may lead to the possibility of measurement bias due to confounding subjective factors. In future research, the safety of rTMS should be quantified using physiological indicators; adding imaging evaluation can obtain more objective and scientific rTMS efficacy; the regulation of rTMS stimulation intensity, pulse number, and intervention time is a new direction for exploring the optimal efficacy of rTMS. Furthermore, due to the limited number of included studies and heterogeneity among the studies, this research could not quantitatively analyze and evaluate the intervention effect of rTMS on related symptoms of Parkinsonian dysarthria. Future studies still need to further improve the rTMS protocol and conduct large-sample randomized controlled trials to further verify the results.

Although this review highlights the overall efficacy of rTMS treatment in improving dysarthria in PD patients, significant heterogeneity exists among the included studies. Differences in study design, such as variations in rTMS frequency, stimulation sites, intervention duration, and participant selection criteria, may contribute to inconsistent outcomes. For instance, some studies targeted motor regions specifically associated with speech production, while others employed broader stimulation sites, potentially influencing efficacy. Additionally, the lack of uniform evaluation metrics, such as inconsistent methods for assessing speech intelligibility and motor control, limits the comparability of results. Potential biases, including small sample sizes, inadequate blinding, and variability in the severity of dysarthria, further complicate the interpretation of findings. Despite these limitations, the results suggest that rTMS can play a role in clinical practice by offering a non-invasive therapeutic option for managing dysarthria in PD patients. However, standardized protocols and larger, well-designed trials are still needed to confirm these findings and guide the development of evidence-based clinical guidelines.

## Data Availability

The original contributions presented in the study are included in the article/supplementary material, further inquiries can be directed to the corresponding authors.
